# A rare case of intradural and extramedullary epidermoid cyst after repetitive epidural anesthesia: case report and review of the literature

**DOI:** 10.1186/s12957-017-1186-4

**Published:** 2017-07-17

**Authors:** Haruki Funao, Norihiro Isogai, Kenshi Daimon, Yuichiro Mima, Hitoshi Sugiura, Takahiro Koyanagi, Masaya Nakamura, Morio Matsumoto, Ken Ishii

**Affiliations:** 1 Department of Orthopaedic Surgery, School of Medicine, International University of Health and Welfare Mita Hospital, Minato, Japan; 2 Spine and Spinal Cord Center, International University of Health and Welfare Mita Hospital, Minato, Japan; 3 Department of Orthopaedic Surgery, Kawasaki Municipal Kawasaki Hospital, Kawasaki, Japan; 4 Department of Pathology, Kawasaki Municipal Kawasaki Hospital, Kawasaki, Japan; 5 Department of Orthopaedic Surgery, Keio University School of Medicine, Shinjuku, Japan

**Keywords:** Intradural extramedullary spinal cord tumor, Epidermoid cysts, Conus medullaris, Epidural anesthesia

## Abstract

**Background:**

Spinal epidermoid cysts are benign tumors, which are rarely seen as an intradural extramedullary spinal cord tumor in the conus medullaris region. Acquired spinal epidermoid cysts are mostly caused by iatrogenic procedures, such as lumbar puncture, and the majority of acquired spinal epidermoid cysts have been reported below the L1 level, because lumbar puncture is usually performed around the iliac crest. Here, we report an extremely rare case of an epidermoid cyst that occurred as an intradural and extramedullary spinal cord tumor attached to the conus medullaris after repetitive epidural anesthesia.

**Case presentation:**

A 67-year-old female presented with a low back pain and left sciatica. Although the patient had experienced occasional mild low back pain for several years, her low back pain markedly worsened 2 months before her visit, as well as newly developed left sciatica resulting in intermittent claudication. She had a history of several abdominal surgeries. All abdominal procedures were performed under general anesthesia with epidural anesthesia in her thoracolumbar spine. Magnetic resonance imaging of her lumbar spine demonstrated an intradural extramedullary spinal cord tumor at the T12–L1 level. Because her symptoms deteriorated, the tumor excision was performed using microscopy. Histological examination of the specimens demonstrated that the cyst walls lined with stratified squamous keratinizing epithelium surrounded by the outer layer of collagenous tissue with the absence of skin adnexa. A diagnosis of epidermoid cysts was confirmed. Her MRI showed complete resection of the tumor, and there was no recurrence at 2-year follow-up.

**Conclusions:**

In this case report, epidermoid cells might be contaminated into the spinal canal during repetitive epidural anesthesia. The patient was successfully treated by complete resection, and there was no recurrence at 2-year follow-up with a good clinical outcome. However, long-term follow-up is required for a potential risk of tumor recurrence.

## Background

Epidermoid cysts (ECs) are benign tumors, which are commonly observed in the intracranial region. Cruveilhier originally named ECs as “tumeurs perlées (pearly tumors)” in 1829, because they had a pearl-like appearance. Although ECs rarely occur in the intraspinal region accounting for less than 1% of all primary spinal cord tumors [1, 2], spinal ECs may cause spinal cord or nerve root compression enlarging the spinal canal.

Since Chiari firstly reported an intramedullary EC in 1883 [3], over 100 cases of spinal ECs have been reported in the literature [4, 5]. It has been reported that the etiologies of spinal ECs are both congenital and acquired. Congenital spinal ECs are thought to have arisen from aberrant ectodermal cells during closure of the neural tube in the embryonic period, and spinal ECs in the conus medullaris region were reported to occur as an intramedullary tumor [5–7]. Spina bifida, spinal dysraphisms, scoliosis and cutaneous/dermal defects are often associated with congenital ECs [2, 7–9]. In contrast, acquired spinal ECs are mostly caused by trauma or iatrogenic procedures, such as lumbar punctures [10, 11]. The majority of acquired spinal ECs had been reported as intradural and extramedullary tumors in the region of the cauda equina (below the L1 level), because lumbar punctures are usually performed around the level of the iliac crests. To the authors’ best knowledge, this is the first report of acquired spinal EC occurring as an intradural and extramedullary tumor attached to the conus medullaris after repetitive epidural anesthesia.

## Case presentation

**Fig. 1:**
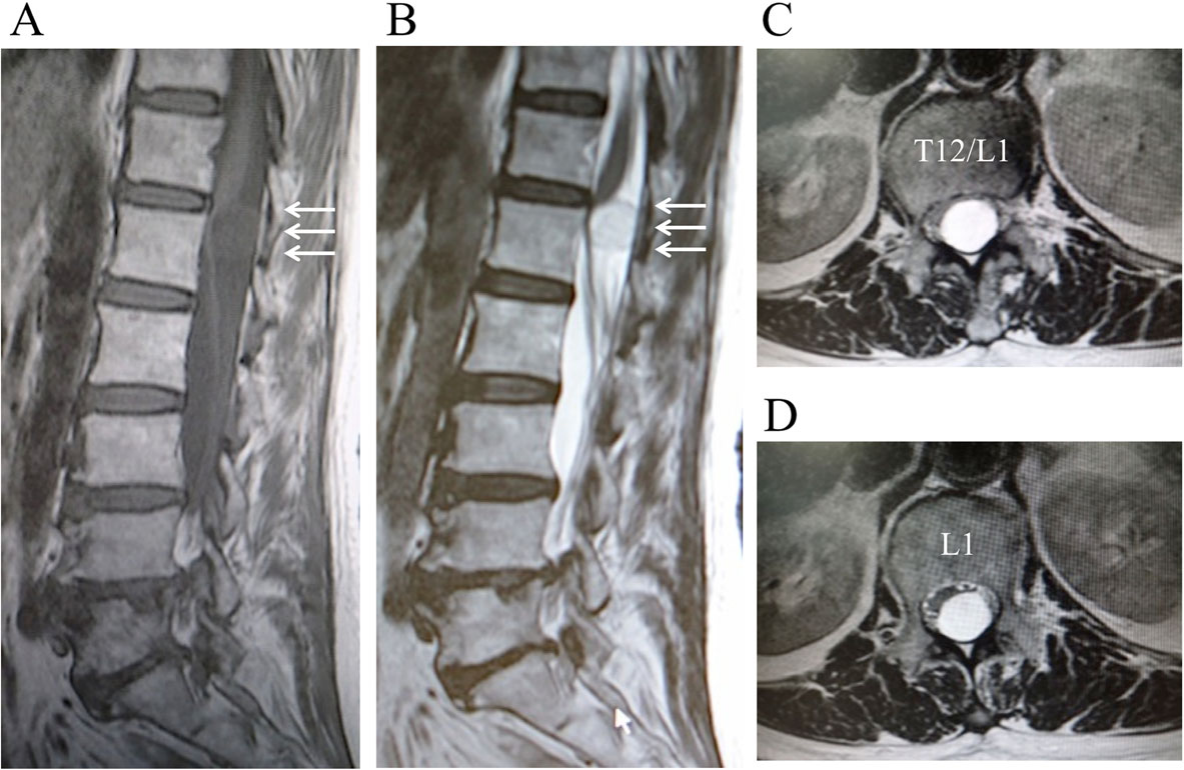
Magnetic resonance imaging (MRI) of the thoracolumbar spine preoperatively. MRI of the thoracolumbar spine showing an intradural extramedullary spinal cord tumor at the T12–L1 (*white arrows*). Views: **a** T1-weighted sagittal, **b** T2-weighted sagittal, **c** T2-weighted axial at T12–L1, and **d** T2-weighted axial at L1. The tumor was hypo-intense on a T1-weighted view and hyper-intense on a T2-weighted view. The spinal cord was markedly shifted anteriorly by the tumor

**Fig. 2:**
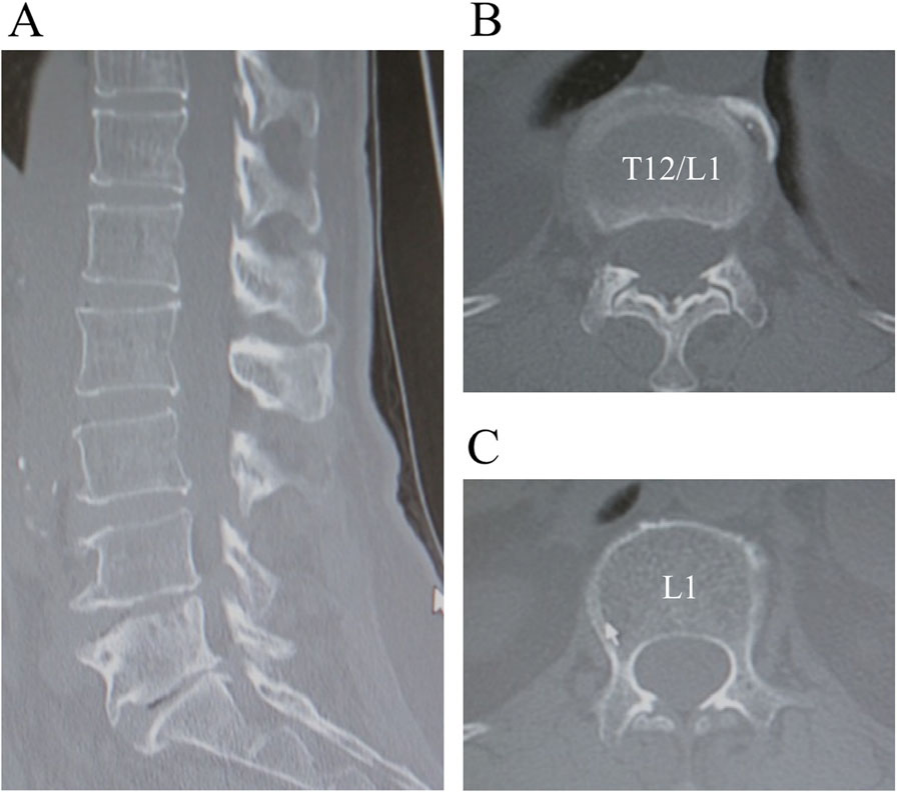
Computed tomography (CT) of thoracolumbar spine. CT of the thoracolumbar spine showing **a** sagittal, **b** axial at T12–L1, and **c** axial at L1 views. There were no specific findings with regard to the tumor, and there was no calcification in the spinal canal

A 67-year-old female presented with a low back pain and left sciatica. Although the patient had experienced occasional mild low back pain for several years, her low back pain markedly worsened 2 months before her first visit to our service. She also complained of newly developed left sciatica during this period, resulting in intermittent claudication. She had not noticed her urinary symptoms specifically, until she was asked at the examination, but she stated that those symptoms had started several months before her first visit. She denied any history of trauma, infectious diseases, or surgeries related to her spine. She had a history of several abdominal surgeries: cholecystectomy for gallstone and Hartmann’s procedure for rectal cancer 10 years ago and Miles’ surgery for anal cancer 9 years ago. She also had a mesh repair surgery for ventral hernia 5 years ago. All abdominal procedures were performed under general anesthesia with epidural anesthesia in her thoracolumbar spine. Radiographs of her lumbar spine showed some degenerative changes including decreased disc height and mild scoliotic changes. Magnetic resonance imaging (MRI) of her lumbar spine demonstrated an intradural extramedullary spinal cord tumor at the T12–L1 level (2.1 cm × 1.2 cm), and her spinal cord was markedly shifted anteriorly by the enlarged tumor (Fig. 1a–d). Computed tomography (CT) of her thoracolumbar spine did not show any calcification in the spinal canal (Fig. 2a–c). Because she had a history of contrast dye anaphylactic shock, a myelography was not performed. She also denied taking an MRI with contrast.

Spinal dysraphism or skin abnormalities were not observed in her lumbar/sacral region. On her neurological examination, she showed full strength and an unremarkable sensory deficit in bilateral upper extremities. The patient showed motor weakness 4/5 in the left lower extremity (iliopsoas, hamstring, quadriceps, foot dorsiflexion, and plantar flexion). Decreased sensation in the left L1 to L3 (6/10) and the left L4 to S1 (8/10) distribution was observed. The right lower extremity demonstrated full strength, and her sensation was intact in the right lower extremity. Although sphincter tone was not diminished and perianal sensation was intact, she was found to have urinary symptoms, such as urinary frequency and a feeling of residual urine. Reflexes were normal in the upper extremities bilaterally; however, hyperreflexia was observed in the patellar reflex bilaterally. Babinski sign was negative bilaterally. Because her symptoms deteriorated, a surgical treatment was performed.

**Fig. 3:**
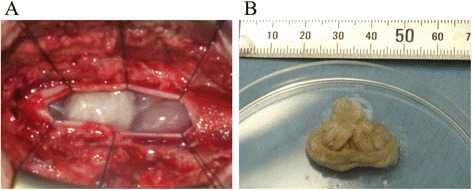
Intraoperative photographs. The tumor excision was performed using microscopy. **a** The tumor was exposed after a laminectomy from T12 to L1 and an incision of the dura mater. **b** The spilled tumor contents were removed as well as the tumor itself. The thin capsule attached to the conus medullaris was also removed carefully

**Fig. 4:**
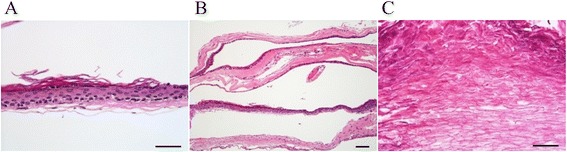
Histological findings. Histological examination demonstrated that **a** the cyst walls lined with stratified squamous keratinizing epithelium (HE, Bar = 50 μm), **b** surrounded by the outer layer of collagenous tissue with the absence of skin adnexa (HE, Bar = 100 μm). **c** Abundant keratin material was also observed (HE, Bar = 100 μm). A diagnosed of epidermoid cysts was confirmed

**Fig. 5:**
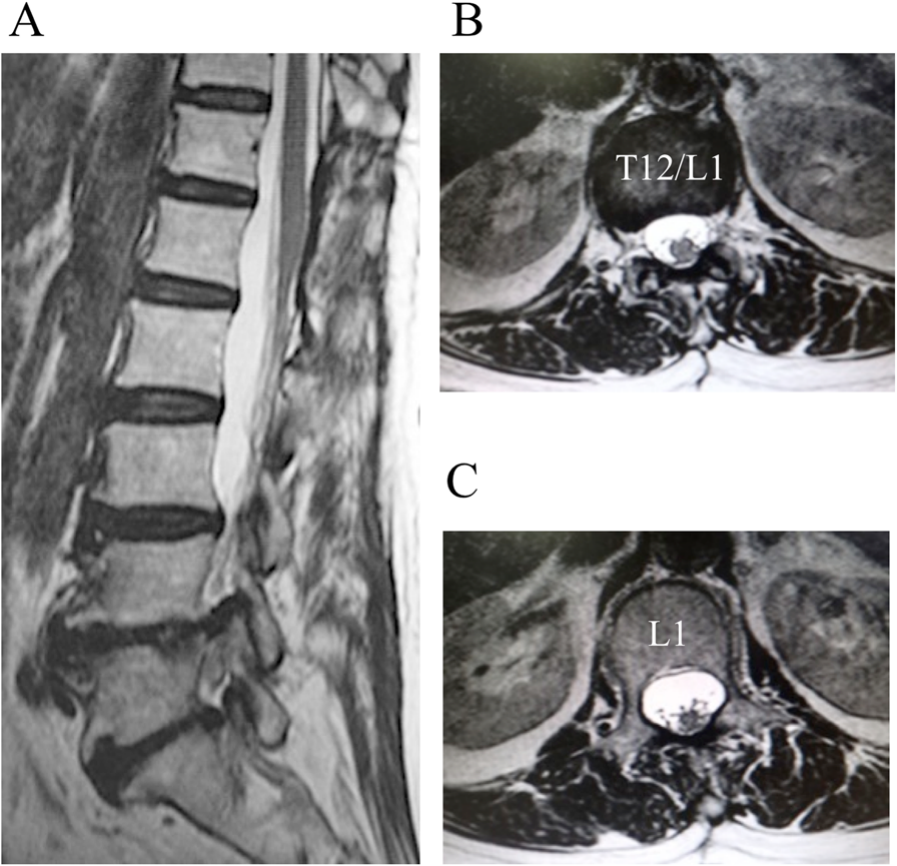
Magnetic resonance imaging (MRI) of the thoracolumbar spine 2 years after surgery. MRI of the thoracolumbar spine obtained 2 years after surgery showing complete resection of the tumor, and there was no recurrence of the tumor. Views: **a** T2-weighted sagittal, **b** T2-weighted axial at the T12–Ll, and **c** T2-weighted axial at L1

A laminectomy from T12 to L1 was performed, and the local dura mater was incised in the midline until the tumor was exposed (Fig. 3a). The tumor excision was performed using microscopy. There was a mild adhesion between the tumor and the arachnoid membrane. Lumbar spinal nerve roots were not involved. Because there was some adherence between the tumor’s thin capsule and the conus medullaris, the capsule ruptured during resection. The spilled tumor contents were removed as well as the tumor itself (Fig. 3b). The thin capsule attached to the conus medullaris was also removed carefully. After the spinal canal was flushed by large amount of water, the dura mater and arachnoid membrane were sutured tightly. The surgical time was 151 min, and the estimated blood loss was 48 ml. Motor evoked potentials were used for neurological monitoring, and there was no alarm during the procedure. Histological examination of the specimens demonstrated that the cyst walls lined with stratified squamous keratinizing epithelium surrounded by the outer layer of collagenous tissue with the absence of skin adnexa. Abundant keratin material was also observed (Fig. 4a–c). A diagnosis of epidermoid cysts was confirmed. She could ambulate immediately postoperatively, and her left sciatica and leg weakness significantly improved 3 months after the surgery. Her MRI showed complete resection of the tumor, and there was no recurrence at 2-year follow-up (Fig. 5a–c).

## Discussion

Since the clinical symptoms of spinal ECs are variable and nonspecific with slow progression, the diagnosis can be delayed. In this case report, the patient visited our clinic 2 months after she noticed her low back pain and left sciatica; however, the majority of patients with spinal ECs usually have long disease duration due to their slow-growing nature [2, 5]. Roux et al. reviewed 47 cases of intramedullary ECs, and they reported that the mean disease duration was 6 years [2]. Morita et al. reviewed the literature of 81 spinal ECs since 1962, and they found that the mean disease duration was 26 months in congenital ECs and 15 months in acquired ECs [5]. Recently, the disease duration seems to be shorter because of the introduction of MRI.

Spinal ECs are difficult to diagnose on clinical symptoms alone. Plain X-rays usually show normal or nonspecific findings, especially in acquired spinal ECs. Penisson-Besnier et al. firstly reported an intramedullary EC diagnosed by MRI [3]. MRI is useful for the diagnosis of spinal ECs, demonstrating that the tumor is hypo-intense on the T1-weighted view and hyper-intense on the T2-weighted view. However, it is sometimes difficult to detect small cysts, because spinal ECs show the same signal as the cerebrospinal fluid on MRI [12, 13]. Myelography can also be performed to evaluate the deviation of the spinal cord, intradural filling defect, or the blocked subarachnoid space. Histological examination is critical for differentiating ECs from other tumors, especially from a dermoid cyst [2, 14]. A specific feature of the histological finding is a lined stratified squamous keratinizing epithelium surrounded by the outer layer of collagenous tissue with the absence of skin adnexa. Desquamation of keratin from the epithelial lining creates numerous cholesterol crystals [15].

The etiologies of spinal ECs are thought to be both congenital and acquired. Acquired ECs were mostly reported in the region of the cauda equina (below the L1 level) [10, 11], because lumbar punctures are usually performed around the level of the iliac crests. In contrast, epidural anesthesia is usually performed at the thoracolumbar spine for abdominal surgeries. To the authors’ best knowledge, there has been only one report describing acquired spinal EC occurring as an intradural and extramedullary tumor just below the conus medullaris after epidural anesthesia [16]. In our case, epidermoid cells might be contaminated into the spinal canal during repetitive epidural anesthesia, and a spinal EC occurred as an intradural and extramedullary tumor attached to the conus medullaris.

Surgical treatment is required when the patients develop neurological deficits. Complete excision of ECs is the essential of surgical treatment [17]. However, complete resection of the encapsulated tumor is difficult for all cases, because the EC’s capsules are very thin and the tumor often adheres to the arachnoid membrane, spinal cord, or nerve roots. Thereby, subtotal resection is also commonly performed [14, 18–20]. The recurrence rate of spinal ECs was reported from 10 to 29% in the previous literatures [5, 11, 21]. Although ECs are benign tumors, local recurrence is reported especially after subtotal excision [2, 5, 13, 21]. It is suggested that incomplete excision of basal germinal cells of the tumor induce the tumor recurrence [3]. Because the tumor content includes fat and cholesterol, they may also produce an inflammatory reaction leading to meningitis [2, 4, 12, 15]. In our case, complete resection of the tumor’s capsule with emptying of the cyst material was achieved, although the tumor’s capsule ruptured during the procedure. Fortunately, the patient did not develop any inflammatory reactions and neurological deterioration postoperatively. There was no recurrence at 2-year follow-up with a good clinical outcome. However, long-term follow-up is required for a potential risk of tumor recurrence.

## Conclusions

Here, we report the first case of spinal EC, which occurred as an intradural and extramedullary spinal cord tumor attached to the conus medullaris after repetitive epidural anesthesia. Complete resection of the tumor’s capsule with emptying of the cyst material was achieved by microsurgery, and good clinical outcome was obtained. Long-term follow-up is required for a potential risk of the tumor recurrence.

## Abbreviations

EC: Epidermoid cyst
CT: Computed tomography
MRI: Magnetic resonance imaging

## References

[1] Amato VG, Assietti R, Arienta C (2002). Intramedullary epidermoid cyst: preoperative diagnosis and surgical management after MRI introduction. Case report and updating of the literature. J Neurosurg Sci.

[2] Roux A, Mercier C, Larbrisseau A, Dube LJ, Dupuis C, Del Carpio R (1992). Intramedullary epidermoid cysts of the spinal cord. Case report J Neurosurg.

[3] Penisson-Besnier I, Guy G, Gandon Y (1989). Intramedullary epidermoid cyst evaluated by computed tomographic scan and magnetic resonance imaging: case report. Neurosurgery.

[4] Manno NJ, Uihlein A, Kernohan JW (1962). Intraspinal epidermoids. J Neurosurg.

[5] Morita M, Miyauchi A, Okuda S, Oda T, Aono H, Iwasaki M (2012). Intraspinal epidermoid tumor of the cauda equine region: seven cases and a review of the literature. J Spinal Disord Tech.

[6] Chandra PS, Manjari T, Devi BI, Chandramouli BA, Srikanth SG, Shankar SK (2000). Intramedullary spinal epidermoid cyst. Neurol India.

[7] von Bostroem E (1897). Ueber die pialen Epidermoide. Dermoide und Lipome und duralen Dermoide. Zentralbl Allg Pathol.

[8] Lunardi P, Missori P, Gagliardi FM, Fortuna A (1989). Long-term results of the surgical treatment of spinal dermoid and epidermoid tumors. Neurosurgery.

[9] Sarma DP, Carter CF, Weilbaecher TG (1984). Intraspinal epidermoid cyst. J Surg Oncol.

[10] Baba H, Wada M, Tanaka Y, Imura S, Tomita K (1994). Intraspinal epidermoid after lumbar puncture. Int Orthop.

[11] Gardner DJ, O’Gorman AM, Blundell JE (1989). Intraspinal epidermoid tumour: late complication of lumbar puncture. CMAJ.

[12] Munshi A, Talapatra K, Ramadwar M, Jalali R (2009). Spinal epidermoid cyst with sudden onset of paraplegia. J Cancer Res Ther.

[13] Teo BT, Lin CC, Chiou TL, Chen SC, Yen PS (2006). Unusual magnetic resonance characteristics of a cerebellopontine angle epidermoid cyst with upper cervical spinal canal extension. J Clin Neurosci.

[14] Kumar A, Singh P, Jain P, Badole CM (2010). Intramedullary spinal epidermoid cyst of the cervicodorsal region: a rare entity. J Pediatr Neurosci.

[15] Er U, Yigitkanli K, Kazanci A, Bavbek M (2006). Primary lumbar epidermoid tumor mimicking schwannoma. J Clin Neurosci.

[16] Manzo G, De Gennaro A, Cozzolino A, Martinelli E, Manto A (2013). DWI findings in a iatrogenic lumbar epidermoid cyst. A case report. Neuroradiol J.

[17] Yin H, Zhang D, Wu Z, Zhou W, Xiao J (2014). Surgery and outcomes of six patients with intradural epidermoid cysts in the lumbar spine. World J Surg Oncol.

[18] Scarrow AM, Levy EI, Gerszten PC, Kulich SM, Chu CT, Welch WC (2001). Epidermoid cyst of the thoracic spine: case history. Clin Neurol Neurosurg.

[19] Fereydoonian NA, Bakhti S, Fereshtehnejad SM, Tabibkhooei AR (2013). Intramedullary thoracic spine epidermoid cyst with myelopathic presentations: a report of a rare case. Clin Neurol Neurosurg.

[20] Gonzalvo A, Hall N, McMahon JH, Fabinyi GC (2009). Intramedullary spinal epidermoid cyst of the upper thoracic region. J Clin Neurosci.

[21] Tang L, Cianfoni A, Imbesi SG (2006). Diffusion-weighted imaging distinguishes recurrent epidermoid neoplasm from postoperative arachnoid cyst in the lumbosacral spine. J Comput Assist Tomogr.

